# Message Framing Effects on Helping Behavior as a Function of Age in Early Childhood and School-Age Children

**DOI:** 10.3390/bs15060758

**Published:** 2025-06-01

**Authors:** Heesun Chang, Hohyun Kim

**Affiliations:** 1 Department of Early Childhood Education, Hyejeon College, Hongseong-gun 32244, Republic of Korea; chhs@hj.ac.kr; 2 Department of Early Childhood Education, Gwangju University, Gwangju 61743, Republic of Korea

**Keywords:** helping behavior, message framing (gain, loss, no frame), issue relevance, preschool and early school-age children

## Abstract

This study determined the effects of message framing on helping behavior as a function of age in preschool and early school-age children. After validating the instrument used in a preliminary study, the researchers conducted a repeated-measures ANOVA of the responses of 90 children aged 5, 6, and 7 years. The results showed that, first, perceived importance and behavioral intention to help tended to increase with age. The results revealed significant differences in both variables depending on the self-relevance of issues and framing type. Second, when issue relevance was high, helping behavior was perceived as more important regardless of framing type, but when issue relevance was low, gain and loss frames were effective. Third, an interaction effect between age, issue relevance, and framing type was identified. While no differences in framing type were found for high-relevance issues, framing effects differed by age for low-relevance issues: loss and gain frames were more effective for younger (5 years) and older children (6 and 7 years), respectively, in promoting behavioral intentions. This study suggests that the effectiveness of message framing depends on the child’s age and issue relevance, which has implications for the development of effective persuasive message delivery strategies for promoting helping behavior among children.

## 1. Introduction

Traditionally, parents and teachers have taught children prosocial behaviors with imperatives such as “help a friend” and “help someone in need”. This approach has shown limited success in encouraging children to voluntarily practice helping behaviors. In Korea, the age of five is known as the “hateful five”—a period of growing autonomy and independence when rebellious attitudes increase, making it difficult to induce behavior change through simple instructions or prescriptive teaching. As children progress into the first and second grades, they develop moral reasoning and a deeper understanding of social relationships. However, simplistic prescriptive approaches are generally ineffective in modifying behavior at this stage of development (Arthur, 2021). Therefore, developmentally appropriate persuasive strategies must be designed to help young children recognize the significance of prosocial behaviors and actively engage in them.

Helping behaviors are the foundation of social relationships and moral development (Bar-Tal, 1982; Eisenberg, 1986). Positive interventions from an early age are important (Izard, 2002) because the environment and experiences of early childhood, a particularly sensitive period, influence whether helping behaviors emerge in adulthood (Miller & Hastings, 2020). However, the complex mechanisms behind developmental changes in helping behavior defy a general approach. During early childhood, as cognitive control becomes available, the ability to put aside one’s own desires for the sake of others gradually emerges (Svetlova et al., 2010). In other words, as children move from self-centeredness to other-centeredness, self-interest and sociality coexist, resulting in helping behavior that is often inconsistent and subject to variation depending on age and situational context. For this reason, the development of helping behaviors in children transitioning from kindergarten to elementary school, when cognitive development is dramatic, is difficult to predict. Therefore, it is essential for experts to develop strategies that effectively promote helping behavior in children during this transitional stage. This study examines message framing as a potential strategy to elicit helping behavior, taking into account children’s developmental characteristics and contextual factors.

Message framing is a persuasive communication strategy that influences individuals’ attitudes and motivations by highlighting either the benefits or costs of a given behavior (Rothman & Salovey, 1997). Experimental studies have examined whether gain-framed or loss-framed messages are more effective in eliciting specific behavioral responses. Gain-framed messages emphasize the positive outcomes of engaging in a behavior (e.g., “If you do this, you will benefit”), whereas loss-framed messages highlight the negative consequences of not engaging in the behavior (e.g., “If you don’t do this, you will lose”). These framing methods play an important role in changing people’s decisions and behaviors. The effectiveness of message framing can vary depending on a variety of factors. For example, the effectiveness of gain versus loss framing depends on gender, age, message content, context, and the self-relevance of the message (Cherubini et al., 2006; Detweiler et al., 1999; Rothman & Salovey, 1997). Early studies found loss frames to be more motivating than gain frames, but later studies found that the effectiveness of loss frames varied by context (Rothman & Salovey, 1997). For example, loss-framed messages tend to be more effective when the issue is highly self-relevant, whereas gain-framed messages are generally more persuasive when the issue holds low personal relevance (Donovan & Jalleh, 2000; Ganzach & Karsahi, 1995). This is because individuals’ tendencies to avoid losses or seek gains are influenced by the perceived relevance of the issue. Moreover, the persuasiveness of a particular message frame can be moderated by various external factors, such as the recipient’s level of education, emotional state, or cultural background (Keller et al., 2003; Smith, 1996). Meta-analytic reviews have found that the persuasive differences between gain- and loss-framed messages are generally small or statistically insignificant. However, such effects may still vary depending on the type of behavior and contextual factors surrounding message relevance (O’Keefe & Jensen, 2006, 2009).

While most existing studies have concentrated on adults, there remains a significant gap in research concerning young children. Early childhood and the early elementary years represent a period of rapid cognitive, social, and moral development, during which children are likely to process information and respond to persuasive messages in ways that differ substantially from those of adults. In particular, analyzing how children receive persuasive messages and respond to different framing strategies may provide valuable insights into promoting helping behavior during early childhood. Accordingly, this study examined the effects of message framing on helping behavior among preschool children (age 5) and early school-age children (ages 6 and 7). Specifically, we investigated how age, issue relevance, and frame type influence children’s perceived importance of a message and their behavioral intentions. The ultimate goal is to identify developmentally appropriate strategies for effectively delivering persuasive messages to children. These findings may inform the development of educational interventions aimed at fostering helping behavior and promoting the early adoption of prosocial tendencies in children.

Therefore, this study aims to examine how age, issue relevance, and message framing interact to influence children’s perceived importance and behavioral intentions to help. In particular, we investigate whether these effects vary developmentally across age groups (kindergarten to second grade) and whether framing conditions (gain, loss, no frame) are differentially effective depending on issue relevance. This framework allows us to identify not only the main effects but also higher-order interactions among the key variables.

Based on these considerations, the present study formulated the following research questions. 1. What are the main effects of message framing type (gain framing, loss framing) and issue relevance (high relevance, low relevance) on children’s perception of and intention to engage in helping behavior? 2. What are the two-way interaction effects between each pair of variables, (a) age and issue relevance, (b) age and message framing type, and (c) issue relevance and message framing type, on children’s perception of and intention to engage in helping behavior? 3. What are the three-way interaction effects between age (5 years, 6 years, 7 years), issue relevance (high relevance, low relevance), and message framing type (gain framing, loss framing) on children’s perception of and intention to engage in helping behavior?

### 1.1. Development of Helping Behavior

Prosocial helping behavior refers to voluntary actions intended to benefit others without seeking external rewards or personal gain (Bar-Tal, 1982; Eisenberg, 1986). Such behaviors include sharing, comforting, assisting, and cooperating and are considered key indicators of social and moral development in childhood. However, in the early stages of development, children’s helping behaviors often do not align with this definition. Young children are more likely to help when prompted by adults or when tangible rewards are anticipated, rather than acting out of internalized moral principles (Bar-Tal, 1982). This early tendency can be explained through Kohlberg’s theory of moral development, which posits that young children typically operate at the pre-conventional level—Stage 1 (obedience and punishment orientation) and Stage 2 (individualism and reward orientation)—where moral decisions are guided primarily by the desire to avoid punishment or receive benefits (Kohlberg et al., 1983). At this stage, helping behavior functions more as a means to achieve personal goals than as an altruistic response. For example, young children may selectively use helping behavior to gain social approval or increase the likelihood of reciprocity (Grueneisen & Warneken, 2022).

As children grow older, helping behaviors generally become more spontaneous, consistent, and internally motivated. This developmental progression is closely linked to improvements in executive functioning, including cognitive flexibility, working memory, and inhibitory control, which allow children to regulate impulses, delay gratification, and consider others’ perspectives (Hongwanishkul et al., 2016). Furthermore, children gradually internalize moral norms through repeated social interactions and adult modeling, enabling them to act prosocially based on empathy, fairness, and concern for others’ welfare (Bar-Tal, 1982; Kohlberg, 1975). Empirical findings support this trajectory. Research shows that from kindergarten to the early elementary years, children increasingly engage in helping behavior even in the absence of adult direction, rewards, or explicit social rules (Bar-Tal et al., 1980). This shift is associated with the development of autonomous moral reasoning and abstract thinking during middle childhood (Eccles, 1999). By this stage, helping behavior reflects social norms such as reciprocity and responsibility toward others and is more often driven by an understanding of others’ needs (Hay & Cook, 2007).

However, developmental increases in helping behavior are not always linear. Some studies suggest a temporary decline in prosocial tendencies during late childhood. For instance, helping behavior has been found to decrease among older elementary school students in specific contexts, such as when personal costs are involved or when the recipient is unfamiliar (Staub, 1970; Jackson & Tisak, 2001). This may reflect a shift from emotionally driven actions to more deliberative decision-making, in which children weigh contextual factors and potential consequences before engaging in helping behavior. In addition to cognitive and emotional development, cultural and situational contexts significantly influence helping behavior. Children’s prosocial actions tend to increase in settings where social roles and responsibilities are emphasized. For example, older children have shown more frequent and responsible helping in contexts such as household labor and caregiving, especially when adult expectations are present (de Guzman et al., 2005). Moreover, children are more likely to help peers who are sick or vulnerable, even at personal cost, indicating the development of empathetic reasoning and moral responsibility (Flook et al., 2019). In sum, helping behavior in childhood emerges through a dynamic interplay between age-related cognitive and emotional development, the internalization of moral norms, and various contextual influences.

### 1.2. Message Framing

Message framing was first utilized as a persuasive message by Meyerowitz and Chaiken (1987), based on prospect theory (Kahneman & Tversky, 1979), which states that people avoid risky decisions when they expect gains and pursue risky decisions when they expect losses. This study framed a message about breast self-examination and found that messages calling for high-risk behavior were more persuasive when presented in a loss frame. Subsequent research has hypothesized that loss-framed messages are generally more persuasive than gain-framed ones, a pattern attributed to two psychological mechanisms: negativity bias, the tendency to be more sensitive to negative than positive information, and loss aversion, the tendency to perceive losses as more impactful than equivalent gains (Gursoy et al., 2022; Maheswaran & Meyers-Levy, 1990; Millar & Millar, 2000; Tversky & Kahneman, 1981). However, empirical findings regarding the superiority of loss-framed messages have been inconsistent (Rothman et al., 2006). The same persuasive message may yield different outcomes depending on factors such as issue relevance (Bosone & Martinez, 2017; Donovan & Jalleh, 2000; J. Lee & Cho, 2022; Moorman & van den Putte, 2008), the recipient’s age (Masumoto et al., 2020), and the mode of delivery (Garcia-Retamero & Cokely, 2011). To enhance message effectiveness, it is essential to tailor message framing to align with the individual characteristics and situational context of the target audience (Rothman et al., 2020).

Message framing provides a contextual approach to encouraging prosocial behavior. Among various methods for fostering prosociality, empathy-centered approaches often lead to a “now-reverse bias”, where individuals focus solely on the immediate social context, while personality-centered approaches may overlook situational influences. The effective activation of helping behavior, regardless of physical proximity or interpersonal closeness, requires the cognitive elaboration of morality in specific helping situations (Hoffman, 2000). This suggests that, in addition to factors such as empathy or personality traits, moral decision-making critically involves a process of context-sensitive perception and judgment (Street et al., 2001). During the transition from preschool to elementary school, children are more likely to base moral judgments on gain–loss reasoning, aligning with Stage 2 of moral development, in which right and wrong are assessed based on rewards and consequences. Given this developmental tendency, message framing appears to be a suitable strategy for shaping children’s intentions to help others. As helping behaviors often decline during the transition from early childhood to elementary school—a period marked by rapid cognitive development—context-sensitive message framing may serve as an effective pedagogical strategy to sustain and enhance prosocial behavior in children.

This study investigates how age and message framing (gain vs. loss vs. no frame) interact to shape helping behavior intentions among Korean preschool children (age 5) and early school-aged children (ages 6 and 7). By experimentally manipulating the level of issue relevance and the type of message framing, this study aims to identify developmentally appropriate and context-sensitive strategies for enhancing prosocial intentions in early childhood.

### 1.3. Factors That Influence Message Framing: Age

Although message framing has been widely studied across various domains, evidence concerning the moderating role of age has yielded mixed results. In the domain of health communication, some studies suggest that younger adults exhibit greater negative reactivity to loss-framed messages compared to older adults (Liu et al., 2019; Mikels et al., 2016). This heightened sensitivity to loss has been attributed to their relatively limited life experience with actual losses, rendering such outcomes more unexpected and emotionally salient (Depping & Freund, 2011). However, other studies in domains such as gambling suggest a different pattern; for instance, it was found that younger adults outperformed older adults in loss-framed decision-making when anticipating monetary losses (Mikels & Reed, 2009).

Similar inconsistencies have been observed in research involving children. For instance, gain-framed messages—rather than the child’s age—were identified as the primary factor influencing healthy food choices among children aged 6 to 10 (Binder et al., 2020). Likewise, benefit-based messages delivered through audiovisual media significantly enhanced handwashing behavior in first-grade students, suggesting the potential power of positively framed messages in health-related contexts (Improgo et al., 2011). In contrast, loss-framed public service announcements promoting water conservation were found to be more persuasive than gain-framed messages for both second- and fifth-grade students, regardless of age or message theme (S. P. Kim & Kim, 2013). These mixed findings imply that the effectiveness of message framing in childhood may depend less on developmental stage alone and more on contextual factors such as message content, delivery medium, and behavioral domain.

However, other studies have questioned the presumed efficacy of message framing in influencing young children’s behavior. For instance, no significant differences were found between gain-framed, loss-framed, and neutral messages in encouraging apple consumption among kindergarteners (Bannon & Schwartz, 2006). Adding further nuance, the persuasiveness of messages among preschoolers aged three to five was found to increase with age, and loss-framed messages were more effective when delivered through a combination of visual and verbal modalities (B. Kim et al., 2022).

Taken together, these findings highlight that the influence of gain and loss framing on children’s behavior is not consistently moderated by age alone. Rather, the framing effect is shaped by a multifaceted interplay of developmental factors, behavioral domains, contextual relevance, and message delivery methods.

### 1.4. Factors That Influence Message Framing: Issue Relevance

The self-relevance of a message is a variable that influences the perceived importance of helping behavior and behavioral intention. Messages perceived as highly self-relevant are typically associated with increased comprehension, which, in turn, enhances the perceived significance of the message and the likelihood of behavioral engagement. Moreover, such messages often exert stronger persuasive effects when framed in terms of potential losses, as loss framing has been shown to capture attention and trigger deeper cognitive processing (B. Kim et al., 2022).

However, empirical findings related this issue remain inconsistent across studies. The effects of gain- and loss-framed messages promoting childhood immunization have also been found to vary depending on the audience’s level of involvement. Specifically, gain-framed messages were more effective among individuals with low involvement, whereas no significant framing effect was observed among those with high involvement (Donovan & Jalleh, 2000). In a study of the effectiveness of public service announcements regarding water pollution that used gain–loss framing, personal gain/public goods, and informational/imagery framing, no effects were found (Park & Kyung, 2002). In contrast, among adult smokers, loss-framed messages were found to be more effective for individuals with high nicotine dependence and strong intentions to quit, whereas gain-framed messages were more persuasive for those with lower dependence and weaker quitting intentions (Moorman & van den Putte, 2008).

Moreover, loss-framed messages have demonstrated greater effectiveness in both personally relevant contexts (e.g., water saving) and public interest contexts (e.g., water conservation) in a study of second and sixth graders (S. P. Kim & Kim, 2013). Loss-framed messages were effective when delivering highly relevant messages simultaneously in pictures and verbally to preschoolers ages 3, 4, and 5 years (B. Kim et al., 2022). Previous studies have found inconsistent message framing effects depending on the child’s age, issue relevance, gain–loss, and delivery modality. Notably, few studies have examined how prosocial behavior is influenced by message framing in conjunction with contextual variables. It is important to identify the factors that effectively shape children’s perceptions of the importance of helping behavior and their behavioral intentions during the transition from age five to the first (age 6) and second (age 7) grades, a period marked by significant cognitive development.

## 2. Research Method

### 2.1. Participants

This study was conducted after having obtained approval from the Institutional Review Board of Gwangju University (approval no.: 2-10411318-A-N-02-202311-HR-023-03). We explained the purpose and necessity of this study to the participants and their guardians and then obtained their written consent prior to their participation. We informed the participants that they could withdraw from this study at any time if they wished. During this study, we ensured that the purpose was explained to the children in an easily understandable manner. After this study, participants received a gift certificate worth KRW 5000 as compensation.

The participants were 110 children aged 5, 6, and 7 years, attending kindergartens and elementary schools in middle-class areas of Seoul, South Korea. Specifically, we employed a non-probability sampling method—namely, snowball sampling—targeting elementary schools and affiliated kindergartens in the Seoul area. We conducted a preliminary test with 20 children. The main test included 90 children: 30 five-year-olds in kindergarten (13 boys [43%], 17 girls [57%]), 30 six-year-olds (first-grade students in elementary school, 16 boys [53%], 14 girls [47%]), and 30 seven-year-olds (second-grade students in elementary school, 14 boys [47%], 16 girls [53%]).

### 2.2. Procedure

#### 2.2.1. Preliminary Experiment

Before proceeding with the main experiment, we conducted a preliminary experiment to examine the appropriateness of the research tools. The preliminary experiment was conducted with 20 children (7 five-year-olds, 7 six-year-olds, and 6 seven-year-olds). The preliminary experiment aimed to assess whether the persuasive messages and picture cards were at the children’s level of understanding and to check the comprehensibility of the survey questions on the perceived importance and behavioral intention of helping behavior and the 7-point Likert scale response method. The preliminary results showed that the persuasive messages and picture cards were appropriate for the children’s level and that they understood the 7-point Likert scale. Words they did not understand were modified with understandable words (e.g., Reduce carbon emissions → Reduce carbon dioxide).

#### 2.2.2. Main Experiment

Based on the preliminary experiment, we finalized the research design and conducted the main experiment. This experiment was conducted in a quiet, empty room with one-on-one interviews between the researcher and child. Each child was presented with a picture card according to a randomly assigned frame type, followed by a persuasive message. After responding to the message, the children were presented with the Perceived Importance and Behavioral Intention of Helping Behavior Questionnaire and a Likert scale card with numbers 1–7. The researcher read the questions one by one, and the child responded by pointing to the number on the Likert scale card. Each child took approximately 20 min to complete the survey. An overview of the experimental design is provided in Table 1.

### 2.3. Research Instruments

#### 2.3.1. Persuasive Messages and Picture Cards

The persuasive messages presented to the children consisted of gain-, loss-, and no-framing conditions. The message content was based on two issues that children aged 5–7 years would understand. The high-relevance issue was “lending a block to help a friend”, a common helping situation in the classroom, and the low-relevance issue was “reducing TV and gaming time to protect the environment”, a socially important topic that has a difficult-to-perceive direct impact.

We presented the three message frames for each of the two issues verbally and with picture cards. For the high-relevance issue, the verbalization was as follows: “You don’t have enough blocks; you need to give some to your friend who doesn’t have enough blocks”. The picture cards showed a child giving blocks to a friend. The picture of the child sharing blocks was presented separately for boys and girls to ensure that the participants’ gender was not affected. We then presented the gain–loss framing message verbally and with picture cards. In the gain-framed condition, an adult hand stroking a child’s head in praise and an illustration of a child’s head (male/female) were presented with the words, “If you share your blocks, you will be told you are a good child”. In the loss-framed condition, a picture of a child (boy/girl) alone with a block was presented with the words, “If you don’t share, your friends won’t lend it to you next time”. In the no-frame condition, the child’s response was heard without any additional picture cards.

In the low-relevance issue, we explained, “You should cut down on TV and video games”, and presented picture cards of TVs and video games. In the gain-framed condition, we said, “If you do this, carbon dioxide emissions will be reduced and polar bears will be stronger and live longer”, and presented a picture of a healthy polar bear on ice. In the loss condition, we said, “If you don’t do this, too much carbon dioxide will make the ice melt, and the polar bears will be in trouble”, and showed a picture of a skinny polar bear on melted ice. In each condition, the child was asked which choice they would make. In the no-frame condition, the child’s answer was heard without any additional picture cards. The high- and low-relevance issues and picture cards were reviewed by two professors of early childhood education and two teachers each from kindergarten and elementary school for content validity. The materials were modified to include age-appropriate language and pictures. The experimental design of this study is shown in Table 1.

#### 2.3.2. Student Report

We used two items to measure the perceived importance of helping behavior and behavioral intentions. We posed questions to capture children’s perceived importance and behavioral intentions regarding the above issues. Regarding the measurement of the perceived importance of helping behavior, the question used was “How important do you think this story is?” The children responded on a 7-point scale (1 = not at all important; 7 = very important). Regarding the measurement of the behavioral intentions of performing the helping behavior, the questions used were “How much do you want to do the action of giving away blocks?” and “How much do you want to do the action of cutting down on TV or video games?” The participants indicated their willingness to help on a seven-point scale (1 = not at all willing; 7 = very willing).

### 2.4. Overview of Analysis

We conducted basic statistical analyses using IBM SPSS^®^ version 25. We calculated the means and standard deviations of each variable. To examine the effects of the independent variables on children’s perceived importance and behavioral intentions toward helping behaviors, we performed a repeated-measures analysis of variance (ANOVA). This analysis allowed for the investigation of the main and interaction effects of age, message frame type, and issue relevance on children’s perceived importance and behavioral intentions. Additionally, we conducted post hoc tests to evaluate group differences.

## 3. Results

### 3.1. Descriptive Statistics

Table 2 presents the descriptive statistics for children’s responses regarding the importance of helping behaviors and their behavioral intentions for helping behaviors, analyzed by age, issue relevance, and framing type. Older children demonstrated higher scores for both the perceived importance of helping behavior and their behavioral intentions compared with younger children.

### 3.2. Role of Age, Issue Relevance, and Framing Type in Children’s Perceptions of Importance of Helping Behavior

**Figure 1:**
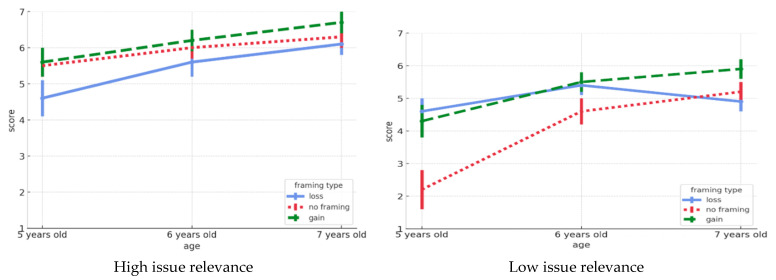
Perceived importance of helping behaviors by age, issue relevance, and frame type.

Figure 1 illustrates the role of age, issue relevance, and frame type in shaping children’s perception of the importance of helping behavior. First, the test of sphericity indicated a violation of the sphericity assumption (*p* < 0.001). Therefore, we applied the Greenhouse–Geisser correction for within-subject effects. Table 3 lists the results of the repeated-measures ANOVA (3 (age) × 2 (issue involvement) × 2 (frame type)) on children’s perceptions of the importance of helping behaviors. We found significant main effects for age (*F* = 21.00, *p* < 0.001), issue involvement (*F* = 49.7, *p* < 0.001), and frame type (*F* = 6.64, *p* < 0.001). Age had a significant effect (*F* = 21.00, *p* < 0.001), indicating that children’s perceptions of the importance of helping behavior varied across age groups. A three-way interaction effect between age, issue relevance, and frame type (*F* = 2.83, *p* < 0.05) was also significant. The effect size for age was η^2^ = 0.241, and for issue relevance, it was η^2^ = 0.266, both indicating large effects. Frame type showed a moderate effect (η^2^ = 0.066), and the interaction between issue relevance and frame type also showed a moderate effect (η^2^ = 0.078). The three-way interaction had a modest effect (η^2^ = 0.079).

### 3.3. Determinants of Children’s Helping Intentions: Age, Issue Relevance, and Framing Type

**Figure 2:**
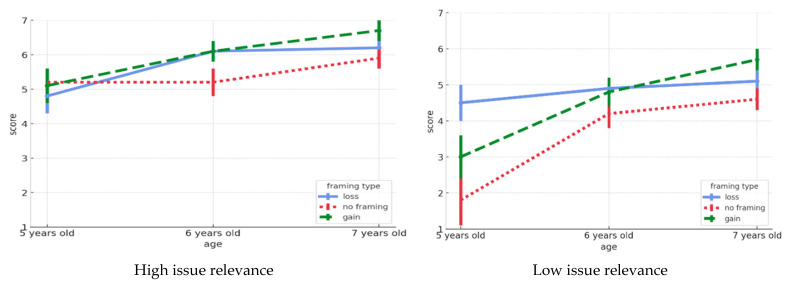
Children’s helping behavior intentions according to age, issue relevance, and message frame type.

Figure 2 illustrates the role of age, issue relevance, and frame type in shaping children’s perception of helping intentions. First, the test of sphericity indicated a violation of the sphericity assumption (*p* < 0.001). Therefore, we applied the Greenhouse–Geisser correction for within-subject effects. We conducted a repeated-measures ANOVA with a 3 (Age) × 2 (Issue Relevance) × 2 (Framing Type) factorial design to examine the effects on children’s behavioral intentions for helping. The results are summarized in Table 4. The analysis revealed statistically significant main effects for age (*F* = 18.10, *p* < 0.001), issue relevance (*F* = 49.47, *p* < 0.001), and framing type (*F* = 5.98, *p* < 0.01). Significant interaction effects further highlighted the complex interplay among these variables. The three-way interaction effect (*F* = 3.56, *p* < 0.01) indicates that the combined effects of age, issue relevance, and framing type are interdependent. For example, older children demonstrated stronger responses to loss-focused framing when the issue was highly relevant, whereas younger children were more responsive to gain-focused framing regardless of issue relevance. The effect size for age was η^2^ = 0.223, and that for issue relevance was η^2^ = 0.282, both reflecting large effects. Frame type showed a moderate effect (η^2^ = 0.090), and the three-way interaction showed a moderate-to-large effect (η^2^ = 0.101).

## 4. Discussion

### 4.1. Effects of Age

The findings of the present study indicate that both the perceived importance of the issue and the behavioral intention to help tend to increase with age. This finding aligns with prior research that indicates that age-related advancements in perspective-taking, causal reasoning, and social responsibility contribute to more sophisticated prosocial behavior (Eisenberg et al., 2006; Garon et al., 2008; Paulus, 2020). These developmental changes are attributed to the internalization of moral values, whereby children increasingly base their helping behaviors on their moral self-concept and personal beliefs rather than extrinsic rewards or reinforcement.

However, when message framing is introduced as a variable, the relationship between age and prosocial behavior does not necessarily follow a linear progression. Although loss-framed messages have been shown to influence behavior across different age groups (S. P. Kim & Kim, 2013), other studies suggests that such framing strategies may have limited effectiveness among young children (Bannon & Schwartz, 2006). Such inconsistencies in previous studies indicate that children’s responses to message framing cannot be explained by age alone but are instead shaped by a variety of factors, including contextual influences, message delivery methods, and their prior experiences (B. Kim et al., 2022). This study’s findings underscore the importance of designing educational programs that foster prosocial behavior in children while considering their developmental stages (Hepach et al., 2013; Malti & Krettenauer, 2013). Furthermore, message framing strategies should be tailored not only to age-related cognitive abilities but also to children’s individual experiences, cultural backgrounds, and levels of cognitive development (Cairns et al., 2013; Lapierre, 2019).

Overall, this study highlights the necessity of integrating multiple factors, including age and developmental characteristics, when designing message framing strategies to enhance prosocial behaviors in children. By incorporating these considerations, educators and policymakers can establish a more effective foundation for the development of prosocial behavior education programs (Durlak et al., 2011; Jones et al., 2017).

### 4.2. Effects of Issue Relevance

The findings of the present study indicate that the perceived importance and behavioral intention to engage in prosocial actions increase when message content is more personally relevant. This result aligns with previous research suggesting that the activation of central cognitive pathways facilitates the rapid integration of information and reinforces behavioral intentions, especially in contexts that are familiar or have been encountered before (Killen & Rutland, 2011). However, the effectiveness of message framing may vary depending on whether the message is presented in a loss or gain frame. While self-relevant messages generally enhance helping behavior intentions, the presence of loss- or gain-framed messages can moderate this effect (B. Kim et al., 2022). For instance, one study found that loss-framed messages were more effective in low-relevance contexts (S. P. Kim & Kim, 2013), whereas another study reported that gain-framed messages yielded stronger effects when self-relevance was low (Donovan & Jalleh, 2000). Additionally, some research suggests that loss framing is particularly effective when high-relevance messages are presented both visually and verbally (B. Kim et al., 2022). Contrarily, some public service announcement studies have found no significant framing effects (Park & Kyung, 2002). These inconsistencies suggest that various contextual factors, such as age, delivery mode, and issue salience, must be taken into account in the design of effective message framing interventions. Nonetheless, the observed increase in behavioral intention to help under high-relevance conditions is likely attributable to heightened attention and deeper cognitive processing (S. P. Kim & Kim, 2013; B. Kim et al., 2022).

These findings underscore the importance of enhancing personal relevance in order to foster prosocial behavior. Specifically, linking distant social issues—such as war, endangered species conservation, and environmental protection—to children’s daily lives may enhance engagement. Experiential learning strategies, including hands-on activities, storytelling, and role-playing, have been identified as effective methods for promoting prosocial development (Gross et al., 2017). While existing research has not fully explored the interplay between self-relevance, prosocial behavior, and message framing in early childhood and lower elementary school children, the present findings suggest that establishing connections between message content and children’s lived experiences is a critical component of effective intervention strategies.

### 4.3. Effects of Framing Types

The findings of this study indicate significant differences in children’s perceived importance and behavioral intention to help, depending on the type of message framing. Specifically, gain-framed messages were more effective for toddlers, whereas the influence of loss-framed messages became more pronounced with increasing age. This age-dependent framing effect may be attributed to developmental changes in cognitive processing that occur from early childhood to the early elementary school years. This can be attributed to the developmental characteristics of young children in Stage 2 of moral judgment, which is primarily based on reward and punishment (Hoffman, 2000; Street et al., 2001). At this stage, gain-framed messages that emphasize positive outcomes and rewards are particularly effective, as children tend to respond emotionally and intuitively (Gamliel & Peer, 2010). At this stage, gain-framed messages that emphasize positive outcomes and rewards are more effective in encouraging prosocial behavior (Gamliel & Peer, 2010). In contrast, older children, who exhibit more advanced cognitive processing abilities, are better equipped to understand the consequences of losses, making loss framing a more persuasive approach (Reyna & Ellis, 1994). These findings align with prior research suggesting that gain framing is particularly suitable for younger children in the context of prosocial behavior, whereas loss framing is more effective for older children, who are more likely to recognize negative consequences and assume social responsibility (B. Kim et al., 2022; S. P. Kim & Kim, 2013).

The results of this study highlight the importance of developmentally appropriate message framing strategies in promoting prosocial behavior. For younger children, gain-framed messages that emphasize the positive aspects of helping others, such as contributing to the community or assisting a friend, may be particularly effective. Conversely, for older children, loss-framed messages that highlight the negative consequences of failing to help—such as the detrimental impact on oneself, others, and the environment—may foster a stronger sense of social responsibility.

### 4.4. Interaction Effects Between Issue Relevance and Frame Type on Perceived Importance of Helping Behavior

The findings of this study reveal a significant interaction effect between issue relevance and message framing on children’s perceived importance of helping behavior. Specifically, when issue relevance was high, children perceived helping behavior as important regardless of the type of message frame. This suggests that intrinsic motivation and personal interest are the primary factors driving engagement in these contexts (A. Y. Lee & Aaker, 2004). Conversely, when issue relevance was low, both gain- and loss-framed messages were more effective than messages without framing. This finding indicates that even when a topic holds low personal relevance for children, it can be perceived as more personally significant when presented within a gain- or loss-framed context.

These results support prior research that suggests that the effects of message framing are more pronounced when issue relevance is low (Block & Keller, 1995). However, this finding contrasts with studies indicating that loss-framed messages are generally more persuasive when issue relevance is high (Donovan & Jalleh, 2000). While research on adults has attributed greater sensitivity to loss-framed messages to risk aversion and heightened reactivity to negative outcomes (Tversky & Kahneman, 1981; Baumeister et al., 2001), meta-analyses have shown that framing effects can vary depending on the message topic and behavioral context (O’Keefe & Jensen, 2009). Additionally, some studies suggest that framing effects differ based on an individual’s regulatory focus (Updegraff & Rothman, 2013). The present study extends this body of research by demonstrating that the interaction between issue relevance and message framing is also evident in a child development context.

Given these findings, message framing strategies should be strategically employed for topics with low relevance to enhance engagement (Masumoto et al., 2020; Rothman et al., 2020). In contrast, for issues with high relevance, emphasizing the intrinsic importance of the topic itself may be more effective than relying on framing techniques (Cesario et al., 2013; Sherman et al., 2006). Furthermore, the design of persuasive messages should be tailored to account for both children’s cognitive developmental stages and the relevance of the issue being addressed (Rothman et al., 2006; Nan, 2012).

### 4.5. Interaction Effects Between Age, Issue Relevance, and Frame Type

The findings of this study demonstrate a significant interaction between age, issue relevance, and message framing in shaping children’s perceived importance and behavioral intention to help. Specifically, for high-relevance issues, both perceived importance and behavioral intentions generally increased with age across all framing conditions (gain, loss, and no frame). However, when high-relevance issues were presented using a loss frame to younger children (age 5), perceived importance and behavioral intentions were notably lower. In contrast, for low-relevance issues, the effects of message framing varied by age. Older children (grades 1–2) exhibited the highest perceptions of importance and behavioral intentions when low-relevance issues were framed as gains, whereas younger children (kindergarten) displayed the highest behavioral intentions when these issues were framed as losses. These results suggest that while both message framing and issue relevance are important factors in shaping children’s responses, their effects become more pronounced when considered within the developmental context of age.

Notably, younger children (age 5) demonstrated lower behavioral intentions to help when high-relevance issues were framed as losses. This phenomenon may be attributed to psychological resistance to loss-framed messages (H. K. Lee & Lee, 2017), reflecting a trait sometimes described as “the hateful five”, where children resist engaging in behaviors when they anticipate a personal loss. Conversely, the increased behavioral intention observed when a loss frame was applied to low-relevance issues may stem from an inherent psychological need to avoid losses, even in cases where the topic itself is not of intrinsic interest. These findings challenge previous research that suggests that the effectiveness of loss-framed messages increases with age (A. M. Lee et al., 2023) and that loss framing is effective regardless of issue relevance (S. P. Kim & Kim, 2013). However, they align with studies indicating that gain-framed messages are generally more effective than loss-framed messages for children in the early elementary grades (Randle et al., 2016; Reinhart et al., 2007).

These results underscore the complexity of message framing effects, which cannot be solely explained by age or issue relevance but instead emerge from the interaction of these factors. Prior research in child development suggests that cognitive and emotional development influences how information is processed (Piaget & Inhelder, 1969; Vygotsky, 1978), which in turn affects message processing and responses (Flavell, 1985; Siegler, 1996). More specifically, message framing effects are closely linked to the development of cognitive and emotional processing abilities across different developmental stages (Reyna & Farley, 2006; Thompson, 1994). This perspective is crucial for understanding how issue relevance and message framing interact in shaping children’s prosocial decision-making.

Overall, these findings emphasize the necessity of an integrative approach to studying children’s prosocial behavior development—one that accounts for the complex interplay of age, issue relevance, and message framing rather than focusing on single-variable effects. This study underscores the need for a contextualized approach to message design aimed at fostering children’s helping behaviors. While message framing and issue relevance each contribute to prosocial decision-making, the findings of this study highlight age as a primary factor driving children’s sensitivity to these contextual cues.

### 4.6. Implications

Message framing can be a democratic teaching method for promoting helping behavior. Among the major educational approaches—authoritarian, democratic, and permissive—early childhood education seeks to use the democratic style for ethically justified education, which is characterized by persuasion as opposed to coercion. Loss–gain framing is not a coercive method that relies on force or authority but rather a persuasive strategy that helps children think and judge autonomously (Murphy, 2001; Powers, 2007; Subba, 2014). This autonomy allows children to form their own beliefs and attitudes from the information provided and internalize the motivation to perform helping behaviors, rather than simply following instructions. From this perspective, message framing can be an effective pedagogical tool for helping young children explore the meaning of prosocial behavior on their own, without coercion. In particular, framing techniques offer children the opportunity to go beyond simple normative instruction—by thinking in terms of gains and losses, children can transform stories that are not directly relevant to their lives into more meaningful experiences. The gain- and loss-focused decision-making processes identified in our study are not simply an external control of framing but can be interpreted as part of a developmental process toward the internalization of helping behavior according to self-determination theory (Deci & Ryan, 2013). For children to deeply understand the value of helping behaviors and practice them autonomously, they need to experience an initial phase of considering the positive and negative consequences of their actions and then gradually acting, with proper guidance, based on communal values and self-directed judgments.

Our findings imply the need for educational interventions that provide opportunities for children to reflect on why helping behaviors are important, how their decisions bring meaning and joy to themselves and others, and how they play a role in the community, rather than simply thinking in terms of “helping because there is a gain” or “helping to avoid loss”. Future research and practice will therefore need to consider using message framing as an initial induction strategy for helping behaviors and then coupling it with educational approaches that can gradually facilitate the internalization process.

### 4.7. Limitations and Future Directions

The limitations of this study are discussed below, which we hope to address in future research. First, this study measured the perceived importance of helping behavior and behavioral intention with a single question. Future research should include a variety of items that enable more precise assessment. In particular, using an instrument that separately measures cognitive and emotional motivations for helping behavior would increase the reliability of this study. Second, we selected block lending and the global environment to represent high- and low-relevance issues, respectively. However, a broader range of topics would increase the external validity of the findings. As children’s personal experiences and values may affect the degree of issue relevance, future studies should consider experimental designs that account for individual differences. Third, this study is an experimental study that intended to capture children’s perceptions. However, perceptions may not translate into actual helping behavior. Children’s responses may differ from their actual behaviors because they may have exaggerated the gain–loss messages presented to them. Therefore, future research should increase the validity of the experimental setting and use methods that measure actual behavior (e.g., behavioral observation, long-term intervention studies) to strengthen reliability. Fourth, the results may have been different if the experiment was a group experiment rather than an individual experiment. Children’s prosocial behavior is influenced by their peers and the people around them. In terms of prosocial behavior, children aged 8–11 years are the most susceptible to social influence, and this does not change with age (Foulkes et al., 2018). Indeed, people often make decisions in groups. As such, future research should examine perceptions in group experiments.

Finally, the effects of issue relevance and message framing on children’s perceived importance of helping behavior are likely to be multifaceted, shaped by both developmental stage and social context. Future research should aim to identify consistent patterns across different age groups and situational contexts to inform more effective intervention strategies.

## Figures and Tables

**Table 1:** Persuasive messages by issue and frame.

Issue	Message Type	Gain	Loss
Friendship (High self-relevance)	Centered	[No framing] “You don’t have enough blocks; you need to give some to your friend who doesn’t have enough blocks.”	
	Reason	[Personal gain] “If you share your blocks, you will be told you are a good child.”	[Personal loss] “If you don’t share, your friends won’t lend it to you next time.”
Environment (Low self-relevance)	Centered	[No framing] “You should cut down on TV and video games.”	
	Reason	[Collective gain] “If you do this, carbon dioxide emissions will be reduced and polar bears will be stronger and live longer.”	[Collective loss] “If you don’t do this, there will be more carbon dioxide emissions and the polar bear will starve to death.”

**Table 2:** Descriptive statistics on the perceived importance of children’s helping behavior and behavioral intention according to age.

	Frame	5 Years Old				6 Years Old (1st Grade)				7 Years Old (2nd Grade)				Total			
		High Involvement		Low Involvement		High Involvement		Low Involvement		High Involvement		Low Involvement		High Involvement		Low Involvement	
		M	sd	M	sd	M	sd	M	sd	M	sd	M	sd	M	sd	M	sd
Perceived importance	Loss	4.50	0.90	4.67	1.07	5.75	1.54	5.33	1.27	6.50	0.53	4.90	1.10	5.59	1.41	5.07	1.20
	No frame	5.10	1.10	2.30	1.16	5.97	1.32	4.52	1.35	6.40	0.97	5.20	1.69	5.88	1.27	4.20	1.70
	Gain	5.02	0.23	4.50	1.07	5.92	1.49	5.35	1.55	6.83	0.58	5.42	1.44	6.00	1.30	5.22	1.46
	Total	4.83	0.87	3.83	1.53	5.89	1.43	5.04	1.44	6.59	0.71	5.19	1.40	5.82	1.33	4.82	1.53
Behavioral intentions	Loss	4.50	0.97	4.50	1.08	5.92	1.35	4.63	1.50	6.10	0.99	4.80	1.75	5.64	1.33	4.64	1.45
	No frame	4.89	0.89	1.50	0.84	4.83	2.05	4.10	1.35	5.80	1.23	4.60	2.12	5.07	1.80	3.87	1.75
	Gain	5.13	0.14	2.88	0.99	6.00	1.41	4.42	1.30	6.42	0.79	5.67	1.56	5.93	1.22	4.48	1.59
	Total	4.79	0.78	3.21	1.56	5.54	1.73	4.37	1.38	6.13	1.01	5.06	1.81	5.55	1.50	4.33	1.62

Note. M = mean; sd = standard deviation; *N* = 120. “Loss”, “No frame”, and “Gain” denote message framing conditions. “High involvement” and “Low involvement” indicate levels of participant engagement.

**Table 3:** Perceived importance of helping behavior by age, issue relevance, and frame type.

	SS	df	MS	F	η^2^
Age (A)	78.76	2	39.38	21.00 ***	0.241
Issue Relevance (B)	67.88	1	67.87	47.97 ***	0.266
Frame Type (C)	13.28	2	6.64	6.64 **	0.066
A × B	4.0	2	2.00	1.41	0.021
A × C	6.25	4	1.56	0.83	0.025
B × C	15.819	2	7.91	5.59 **	0.078
A × B × C	16.026	4	4.00	2.83 *	0.079
Error [B]	186.77	132.00	1.42		
Error [between groups]	247.65	132	1.876		

Note. * *p* < 0.05, ** *p* < 0.01 *** *p* < 0.001. SS = Sum of Squares; df = Degrees of Freedom; MS = Mean Square; F = F-ratio; η^2^ = eta squared (effect size).

**Table 4:** Children’s intention to help according to age, issue relevance, and frame type.

	SS	df	MS	F	η^2^
Age (A)	75.11	2.00	37.55	18.10 ***	0.223
Issue Relevance (B)	98.43	1	98.43	49.47 ***	0.282
Frame Type (C)	24.79	2.00	12.40	5.98 ***	0.090
A × B	4.95	2.00	2.47	1.24	0.019
A × C	7.73	4.00	1.93	0.93	0.030
B × C	7.86	2.00	3.93	1.98	0.030
A × B × C	28.28	4	7.07	3.56 **	0.101
Error [B]	250.71	126.00	1.99		
Error [between groups]	261.38	126.00	2.07		

Note. ** *p* < 0.01 *** *p* < 0.001. SS = Sum of Squares; df = Degrees of Freedom; MS = Mean Square; F = F-ratio; η^2^ = eta squared (effect size).

## Data Availability

All the raw data are available from the first author upon request.
